# Cervical cancer knowledge and screening behaviors among female university graduates of year 2012 attending national graduate orientation program, Bhutan

**DOI:** 10.1186/1472-6874-14-44

**Published:** 2014-03-12

**Authors:** Tshering Dhendup, Pandup Tshering

**Affiliations:** 1 Head, Health Research and Epidemiology Unit, Planning and Policy Division, Ministry of Heath, Kawangjangsa, Thimphu, Bhutan; 2 Bhutan Medical and Health Council, Ministry of Health, Kawangjangsa, Thimphu, Bhutan

**Keywords:** Bhutan cervical cancer knowledge, Screening behavior, Determinants, University graduates

## Abstract

**Background:**

Cervical cancer is the leading female cancer in Bhutan. This study describes the level of cervical cancer knowledge and screening behaviors among female university graduates attending the National Graduate Orientation Program (NGOP), 2012.

**Methods:**

A cross-sectional study of female graduates attending NGOP was conducted using self-administered anonymous questionnaire developed through literature reviews and expert discussions to elicit information on demographic characteristics, knowledge, screening behaviors and determinants of cervical cancer. The association of demographic and other important study characteristics with uptake of Pap test was investigated using cross tabulation and Fischer Exact test. Frequencies and percentages were calculated for all the questions.

**Results:**

The average age of the participants was 23.43 ± SD 2.73. About 92% (n = 513) of the respondents were aged 25 years or less and 7.9% (n = 44) of the respondents were aged 26 or more. The study revealed low cervical cancer knowledge and poor screening behavior among the graduates. The mean knowledge score was 3.571 (SD1.75, Range 0–8). About 6% (n=34) of the respondents reported undergoing Pap test at least once and 94% reported as never having done Pap test. The most commonly cited reasons for not doing Pap test included “never thought I needed one” (57%, n = 320), “embarrassment of being examined by male health professional” and “fear of finding out cancer”. The study revealed evidence of significant association between increasing age, those who are married, knowledge score and those recommended for screening by health professionals with the uptake of Pap test.

**Conclusion:**

Our study revealed poor knowledge and screening behaviors among female university graduates in Bhutan. This may be suggestive of even poorer awareness and screening practices among young unmarried women who are less educated or with no education. Although our study group is not appropriate for measuring practice of cervical cancer screening in the country, the findings are expected to highlight the shortcomings and trigger development of comprehensive cervical cancer control programs in Bhutan.

## Background

Cervical cancer, though largely preventable, is the second most common female cancer internationally and a leading cause of cancer deaths among females in the developing countries [1,2]. In 2010, the global incidence of cervical cancer was 454,000 cases, of which about 50% resulted in death [3]. More than 85% of global cervical cancer deaths occur in low and middle-income countries, [4] reflecting poor control and early detection measures in these countries. In 2008, over 1.1 million people died of cancers in the South East Asia Region [5] of the World Health Organization where Bhutan is a member country. Of these, 35% deaths were due to breast and cervical cancers [5].

Health care services in Bhutan are provided free and solely by the state to its population of 634,982 [6] people through a network of Basic Health Units, District Hospitals, and Regional and National Referral Hospitals. There are currently no private health care providers in Bhutan. When treatment is not available in Bhutan, the state covers the treatment costs of patients in another country. Majority of the cases referred outside Bhutan comprise of non-communicable diseases like cancers. Cervical cancer is the leading female cancer site in Bhutan with an estimated age standardized incidence rate of 12.8 per 100,000 [7]. The age standardized mortality rate for cervical cancer in Bhutan is estimated to be 7.1 per 100,000 which is slightly more than twice that of the United States of America (2.7/100,000) and Japan (2.8/100,000) [7].

A nationwide Pap test program in Bhutan was launched in the year 2000. Prior to this, Pap test was offered as opportunistic screening in the capital city, Thimphu, since 1980 [8]. The American Cancer Society recommends cervical cancer screening at age 21 with repeat tests after every 3 years till the age of 30 years and thereafter, every 5 years with combined HPV(Human Papillomavirus) test till the age of 65 years [9]. The national guideline on cervical cancer screening of the Ministry of Health, Bhutan recommends all women between ages of 20–60 years to undergo Pap test with repeat tests initially after 6 months and then after every 3 years [8]. Pap test is provided free of cost by trained female health assistants (primary health care workers), nurses, and medical doctors through maternal and child health (MCH) clinics in all district hospitals, regional and the national referral hospitals, and in Basic Health Units (BHUs) where trained female health workers are available.

Early detection can greatly increase the chance of successful treatment resulting in approximately 40% reduction in incidence and mortality associated with invasive cancer [10]. The detection of most of cervical cancer cases at a late stage in Bhutan is an indication that the freely provided screening services are not utilized or are poorly utilized. Therefore, education to increase awareness of risk factors and promotion of early detection becomes an imperative.

To our knowledge, currently there are no published data on cervical cancer knowledge and screening practices in Bhutan. This study attempts to describe level of knowledge, current screening behaviors and barriers to screening among female university graduates attending the 2012 National Graduate Orientation Program (NGOP) in Thimphu, the capital city of Bhutan.

## Methods

Every year, graduating Bhutanese students from universities within and outside the country congregate in Thimphu for a period of two weeks to attend NGOP, a program organized by the Ministry of Labor and Human Resources, Bhutan. The certificate of attendance to this program is one of the main prerequisite for employment in both public and private sectors in the country leading to almost 100% attendance rate each year by university graduates across all disciplines. A cross-sectional study of all female graduates attending NGOP was conducted in August, 2012.

Participation was voluntary and written informed consent was obtained. This study was approved by the Research Ethics Board for Health of the Ministry of Health, Royal Government of Bhutan which is a member of Forum for Ethical Research Committees in Asia and the Western Pacific Region (FERCAP).

A structured anonymous questionnaire developed through literature reviews [11-13] and expert discussions was used to elicit information on respondent’s demographic characteristics, cervical cancer knowledge, screening practices and determinants. The response choice for knowledge questions consisted of ‘agree’, ‘disagree’ and ‘don’t know’ options. It took approximately 10–15 minutes to complete the questionnaire. The questionnaire was pre-tested with female students studying postgraduate diploma courses at the Royal Institute of Management in Thimphu and necessary changes were made to the questionnaire.

Respondents were asked about the frequency of Pap test. The frequency of Pap test was dichotomized as “at least once” and “never”. Similarly, the marital status question was dichotomized into “married” and “not married”. Frequencies were calculated for all the questions. Data were entered using Microsoft Excel and coded, cleaned and analyzed using Statistical Package for the Social Sciences (SPSS, Version 20).

A knowledge score was calculated for each participant based on the number of questions correctly answered in the knowledge Section. A score of 1 was assigned to every correct answer and a score of zero to incorrect or “don’t know” responses. Each respondent’s knowledge score was calculated by adding number of correct answers. A maximum score of 8 and a minimum score of 0 could be obtained. A total score of 4 or less were categorized as possessing “below average” knowledge and a score of 5 or more as having “above average” knowledge. The association of age, marital status, whether or not recommended for Pap test by health professionals, and country of study with uptake of Pap test was investigated using cross-tabulation and Fischer Exact test. The significance level was set at p < 0.05.

## Results

Of the total of 923 registered female graduates for the NGOP 2012, 571 participated and completed the study questionnaire. However, available information indicated that there were no major differences in the characteristics of interest such as age, marital status, and country of graduation between the 571 who participated in the study and the remaining who did not. Out of the 571 participants, 559 were eligible for the final analysis after excluding questionnaires with missing data. The demographic and descriptive characteristics of the study participants are shown in Table 1. The mean age of the participants was 23.43 ± SD 2.73. About 92% (n = 513) of the respondents were aged 25 years or less and 7.9% (n = 44) of the respondents were aged 26 years or more. Forty five percent (n = 253) of the respondents graduated from universities/institutes outside Bhutan and 11% (n = 64) reported as married.

**Table 1:** Demographic and descriptive Characteristics of study participants

**Variables**	**Frequency (n)**	**Percent (%)**
**Age**		
≤ 25	513	91.8
≥26	44	7.9
**Mean ± SD 23.43 ± 2.73**		
**Marital status**		
Not married	495	88.6
Married	64	11.4
**Country of graduation**		
Bhutan	300	53.7
Ex-Country	253	45.3
**History of cancer cervical or breast cancer among family and close friends**		
Yes	37	6.6%
No	520	93%

### Cervical cancer knowledge

The knowledge of cervical cancer is shown in Table 2. 53% of the respondents agreed that multiple sexual partners increased the risk of getting cervical cancer and about 53% of the respondents knew sex at an early age as risk factor for cervical cancer. About 26% of the respondents were aware of the history of cervical cancer among close family relatives as a risk factor. Similarly, 25.4% of the respondents were aware of the link between cervical cancer and smoking. While 14.8% of the respondents agreed that testing for cervical cancer is not necessary for those who received HPV vaccination, 38.6% did not know the answer. About 53% of the respondents were aware of the purpose of Pap test.

**Table 2:** Cervical cancer knowledge among female university graduates

**Statements**	**Number**	**Percent**
If you have a close blood relative with cervical cancer, your chance of getting cervical cancer increases		
***Agree***	150	26.8
Disagree	200	35.8
Don’t know	209	37.4
Total	559	100
Testing for cervical cancer is not necessary for those who received HPV vaccination in Bhutan		
Agree	83	14.8
***Disagree***	260	46.5
Don’t know	216	38.6
Total	559	100
Being sexually active from early age increases the risk of getting cervical cancer		
***Agree***	282	50.4
Disagree	84	15.0
Don’t know	193	34.5
Total	559	100
Risk of developing cervical cancer increases with multiple sexual partners		
***Agree***	296	53.0
Disagree	85	15.2
Don’t know	177	31.7
Total*	558	99.8
Smoking tobacco/or exposure to tobacco smoking increases risk of getting cervical cancer		
***Agree***	142	25.4
Disagree	215	38.5
Don’t know	202	36.1
Total	559	100
Poverty is a risk factor for cervical cancer		
***Agree***	123	22.
Disagree	283	50.6
Don’t know	153	27.4
Total	559	100
If diagnosed at early stage, development of cervical cancer can be prevented		
***Agree***	444	79.4
Disagree	15	2.7
Don’t Know	100	17.9
Total	559	100
Pap test is used for detection and prevention of cervical cancer		
***Agree***	300	53.7
Disagree	18	3.2
Don’t know	241	43.1
Total	559	100

*The total reflects only those who responded to the question.

Correct answer to each question is shown in bold italics.

The average knowledge score was 3.57 (SD1.75). Twenty seven graduates (4.8%) obtained a score of 7 and above and 28 (5%) graduates obtained a score of zero. A total of 391 (69.9%) and 168 (30.1%) graduates were found to possess “below average” and “above average” knowledge, respectively.

### Frequency of Pap test and its barriers

As shown in Table 3, about 6% (n=34) of the respondents reported undergoing Pap test "at least once" and about 94% reported as “never”. The most common reasons cited for not doing Pap test included “never thought I needed one” (n = 320, 57.2%) and “embarrassed to get examined by male health professional” (n = 134, 24%). Only 4.1% (n = 23) chose the option “because of past unpleasant experience with health workers” for not doing Pap test. About Ninety five percent (n = 530) reported that they were not recommended for Pap test during their last visit to a health facility in Bhutan.

**Table 3:** Reasons cited for not doing Pap test

**Statements**	**N**	**%**
Never thought that I needed one	320	57.2
Embarrassed to get examined by male health professional	134	24
Afraid to find out cancer	111	19.9
Wasn’t aware such services are available in our health facilities	34	6.1
Because of past unpleasant experience with health professionals	23	4.1
Others	14	2.5

Increasing age (p = 0.000), marital status (p = 0.000), knowledge score (0.023), and whether or not recommended for screening test during previous visit to health facility (0.01) showed evidence of significant association with uptake of Pap (Table 4).

**Table 4:** Cross tabulation of study characteristics with uptake of Pap test

	**Had Pap test (%)**		**P value**
**Variable**	**At least once**	**Never**	
Age in years			
≤ 25	17 (3.3%)	496 (96.7%)	0.000
≥26	17 (38.6%)	27 (61.4%)	
Marital status			
Married	19 (29.7%)	45 (70.3%)	0.000
Not married	15 (3.0%)	480 (97.0%)	
Knowledge score			
Below average	18 (4.6%)	373 (95.5%)	0.023
Above average	16 (9.5%)	152 (90.5%)	
Country of graduation			
Bhutan	14 (4.7%)	286 (95.3%)	0.110
Ex-Country	19 (7.5%)	234 (92.5%)	
Whether or not recommended for pap test and breast examination during the previous visit to health facility in Bhutan			
Yes	11 (50.0%)	11 (50.0%)	0.000
No	23 (4.3%)	507 (95.7%)	

## Discussions

In general, our study revealed poor cervical cancer knowledge of risk factors and detection method among female university graduates of Bhutan. Similar studies conducted in Malaysia [14], South Africa [15], Kolkata [16] and in the United States of America [17,18] reported comparable results. About 53% (n = 353) of the participants were aware of the purpose of Pap test which is much lower than those reported from studies conducted among Taiwanese (80%) [19] and Greek (94%) [20] students. Only about 25% of our study respondents knew about the link between smoking and cervical cancer which is slightly higher than studies reported from Sri Lanka (20.8%) [21], Nepal (0.2%) [21] and Ghana (1%) [22] but much lower than that reported from studies conducted in Malaysia (61%) [23]. On the other hand, our study revealed better awareness of the link between cervical cancer and multiple sexual partners (53%) and early onset of sexual activity (50%). Knowledge of the link between cervical cancer and early onset of sexual activity in our study was found to be higher than that reported from study [21] conducted among female youths in Sri Lanka (27.7%), India (26.1%) and Nepal (38.8%). In health facilities across Bhutan, cervical cancer education is generally provided by health professionals to women visiting MCH clinics (who are mostly married women) for maternal, child health and family planning purposes. However, the absence of a standardized system of disseminating cervical cancer education to women other than those visiting MCH clinics in the country may be attributable to the low awareness of cervical cancer among our study population.

Our study found that about 6% (n=34) of respondents reported doing Pap test "at least once" and 94% (n=525) reported as “never” despite it being freely available in the country. Studies conducted elsewhere yielded similar results of low Pap test uptake. For example, studies among female students in Nigeria [24], Malaysia [23], and Turkey [25] showed low utilization of Pap test. In general, while the poor uptake of Pap test could be explained by the fact that people do not usually undergo health checks without first experiencing health problems, the absence of systematic and active promotion of screening program in the country may be attributable to the low utilization of Pap test. While any woman can voluntarily avail free Pap test services in Bhutan, currently only those who visit MCH clinics are routinely recommended to undergo Pap test. Our young study population, a vast majority of whom are 25 years or younger and unmarried (91%) may explain the low uptake of Pap test. Though the average age at first marriage in Bhutan is 22 years [26], a household survey revealed that 22% of women aged 15–49 in Bhutan were never married/in union [27]. Therefore, there is a need to expand the offer of Pap test by health professionals beyond those who visit MCH clinics to all eligible women visiting health facilities for any purpose.

The majority of our study respondents cited “never thought I needed one” (57%, n = 320), “embarrassment of being examined by male health professional” (24%, n = 134) and “fear of finding out cancer” (20%, n = 119) as the commonest reasons for not doing Pap test. A study conducted among female university students in Japan [28] revealed “embarrassment of being examined by male health professional” as an important barrier to Pap Test. Studies conducted elsewhere [23,29,30] found “embarrassment” as a common barrier to Pap test. Study conducted among 10^th^ graders in the United States of America [31] found fear of cancer (37%) as a barrier to Pap test. In order to overcome the common barriers mentioned, it may therefore be beneficial to emphasize on the availability of trained female health workers to conduct the Pap test and to increase awareness about the recommended age-group for Pap test in all cervical cancer related public awareness/education activities in Bhutan. It is important to note that despite 79% (n = 444) of our study respondents knowing that early detection can prevent development of cervical cancer, the poor screening behavior suggests that factors other than education may play a major role in influencing screening practices. It may be useful to recommend studies exploring socio-cultural and economic factors associated with Pap test in Bhutan. Further, according to the Reproductive Health Program of Bhutan’s Ministry of Health, the transfer of female health workers trained in Pap test procedures to other health facilities may have contributed to the low utilization of Pap test since such transfers leave the health facilities, particularly the Basic Health Units, without trained personnel to conduct Pap test. It may be useful to provide training in Pap test procedures to students undergoing General Nursing Midwifery and Health Assistant courses at the Royal Institute of Health Sciences, the sole training institute for such categories of health workers in the country.

Our study found evidence of significant association (p ≤0.05) between Pap test uptake and those recommended by health professionals for screening during their visit to a health facility. Studies [24,32,33] conducted elsewhere have revealed lack of recommendation by healthcare professionals as one of the main reasons for not undergoing Pap test. This study also showed evidence of significant association with increasing age, those who are married, and knowledge score with the uptake of Pap test.

## Conclusion

Our study revealed poor knowledge and screening behaviors among female university graduates in Bhutan. This may be suggestive of even poorer awareness and screening practices among young unmarried women who are less educated or with no education. Although our study group is not appropriate for measuring practice of cervical cancer screening in the country, the findings are expected to highlight the shortcomings and trigger development of comprehensive cervical cancer control programs in Bhutan.

## Abbreviations

HPV: Human Papillomavirus; MCH: Maternal and child health; NGOP: National Graduate Orientation Program; FERCAP: Forum for ethical review committees in the Asian and Western Pacific Region.

## Competing interests

The author(s) declare that they have no competing interests.

## Authors’ contributions

TD conceptualized and designed the study, analyzed, and interpreted the data, prepared manuscript, gave final approval for the publication and agrees to be accountable to all aspects of this study. PT coordinated and collected data, reviewed study questionnaire, participated in the drafting of manuscript and agrees to be accountable to all aspects of this study. Both authors read and approved the final manuscript.

## Authors’ information

1. Tshering Dhendup

Gender: Male

Date of Birth: 01/01/1974

Country of Birth: Bhutan

Citizenship: Bhutanese

Address: Head, Health Research and Epidemiology Unit, Ministry of Health, Post Box no. 726, Kawanjangsa, Thimphu: Bhutan

Email: tsheringdhendup@health.gov.bt; t_dhendup@hotmail.com Phone: +975-17110708; +975-2-322602 (ext 314)

Education

June 2009 Master of Public Health, National University of Singapore, Singapore

June 2002 B.Sc (Honors) Biomedical Science, University of Wolverhampton, UK

2. Dr. Pandup Tshering

Gender: Male Registrar

Date of Birth: 10/05/1963

Address: Registrar, Bhutan Medical and Health Council, Thimphu, Bhutan

Phone: 17610512(M), E-Mail: pandup_tshering@yahoo.com

Education

2003-2004-Masters in Public Health (Health System Development), University of Chulalongkorn, Bangkok, Thailand

2000-2001-Diploma in Dermatology; Institute of Dermatology, Department of Medical Services; Bangkok, Thailand

1995–1996: General Practice Course, CMC, Vellore

1983-1989- Bachelor of Medicine and Surgery (MBBS); Chittagong Medical College, University of Chittagong, Bangladesh

## Pre-publication history

The pre-publication history for this paper can be accessed here:

http://www.biomedcentral.com/1472-6874/14/44/prepub
